# Performance Optimization of Solar Water Heating Systems Using Molten Salt Thermal Energy Storage Across Varying Tilt Angles

**DOI:** 10.1002/gch2.202500074

**Published:** 2025-07-02

**Authors:** Natrayan Lakshmaiya, Gobikrishnan Udhayakumar, Prem Kumar Reddy M, Karthick M, Ramya Maranan, Prabhu Paramasivam, Asefash Getachew Girma

**Affiliations:** ^1^ Department of Mechanical Engineering Saveetha School of Engineering SIMATS Chennai Tamil Nadu 602105 India; ^2^ Department of Mechanical Engineering Sona College of Technology Salem Tamil Nadu 636005 India; ^3^ Department of Mechanical Engineering Aditya University Surampalem 533437 India; ^4^ Department of Mechanical Engineering Vel Tech Rangarajan Dr. Sagunthala R&D Institute of Science and Technology Chennai Tamil Nadu 600062 India; ^5^ Division of Research and Development Lovely Professional University Jalandhar – Delhi G.T. Road Phagwara Punjab 144411 India; ^6^ Research and Innovation Cell Rayat Bahra University Mohali Punjab 140301 India; ^7^ Department of Chemical Engineering College of Engineering and Technology Mattu University Mettu 318 Ethiopia

**Keywords:** collector efficiency, heat transfer coefficient, inclination angles, molten salt thermal energy storage, solar water heater

## Abstract

This study proposes a novel approach to enhance the performance of solar water heating systems by integrating molten salt thermal energy storage (MSTES) and evaluating its effectiveness under varying tilt angles (15°, 30°, 45°, and 60°). While prior research has extensively explored solar collectors and conventional storage media, there have been limited studies that experimentally assessed the combined effect of MSTES and tilt angle optimization on thermal performance. To address this gap, a parabolic trough collector system is employed using a eutectic mixture of sodium nitrate and potassium nitrate, known for its high thermal stability and energy retention. Key performance metrics, including collector efficiency, heat transfer coefficient, and storage efficiency, are analyzed under different tilt configurations. Results revealed that a 60° tilt angle offered the best performance, achieving a collector efficiency of 75%, a heat transfer coefficient exceeding 880 W m^−^^2^ K, and a storage efficiency of 61% during peak solar radiation. These findings highlight the effectiveness of MSTES in maximizing solar energy absorption and storage, thereby contributing to the development of high‐efficiency solar thermal systems that are adaptable to diverse climatic conditions and energy demands.

## Graphical abstract

A solar water heating system integrated with molten salt thermal energy storage (MSTES) was experimentally evaluated under varying collector tilt angles. Using a parabolic trough collector and sodium‐potassium nitrate salt, the study identified optimal thermal performance at 15° tilt, achieving high collector efficiency, effective heat transfer, and improved storage capacity for sustainable energy utilization.

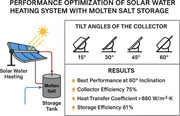

## 1 Introduction

Solar water heaters have long become one of the most progressive and effective methods for utilizing solar energy to satisfy domestic and industrial heating needs. Generally, a solar water heating system comprises solar collectors, a storage tank, and a heat transfer system that circulates the heated fluid.^[^ ^1^ ^]^ Primarily, the heater is designed to absorb solar radiation and convert it into thermal energy to be used for water heating. As a result, the system reduces the use of other non‐renewable conventional fuels and substantially cuts greenhouse gas emissions, thereby preserving the environment.^[^ ^2^ ^]^ Many solar water heater systems rely on thermal energy storage to capture sunlight and retain the heated water for later use on a cloudy day or at night. Many types of materials are used for this purpose, but molten salts, such as nitrate and carbonate salts, appear to be the most effective.^[^ ^3^ ^]^ The main reason is their high heat capacity and thermal stability. They can store very large amounts of thermal energy and release it at high rates when heated. Due to these factors, utilizing molten salt thermal energy storage in solar water heaters may enhance their performance and energy efficiency.^[^ ^4^ ^]^

The inclination angle of the solar collectors, also known as the tilt angle, is one of the most critical characteristics that define the performance of solar water heaters. The inclination angle generally determines the amount of solar radiation exposed to collectors.^[^ ^5^ ^]^ As a rule, the collectors should be placed and adjusted to ensure their maximum exposure to the sun at a direct, perpendicular angle. Notably, because there are differences in the duration and intensity of solar radiation throughout the year, as well as between regions, the ideal angle of collectors during the day and year should not be the same.^[^ ^6^ ^]^ As such, the inclination angle of the solar collector directly determines the amount of solar radiation collected, resulting in heating and storage, and utilizing thermal energy to provide a hot water supply.^[^ ^7^ ^]^ Molten salts are a reliable solution for thermal energy storage due to their favorable thermo physical properties. They can operate at sufficiently high temperatures, allowing for the efficient heating and storage of solar energy.^[^ ^8^ ^]^ The fact that molten salts remain liquid within a broad temperature range ensures very stable thermal conductivity and relatively small heat losses. Furthermore, molten salts possess high specific heat capacity, which allows for the storage of considerable energy per unit volume.^[^ ^9^ ^]^

Past research studies focusing on solar water heaters have indicated that these heating devices effectively harness solar energy for heating applications in various residential and industrial settings.^[^ ^10^ ^]^ Early studies concentrated especially on describing the design and operation of the two most common types of heating devices: the flat‐plate collector and the evacuated tube collector. Various materials and complex arrangements have been used to improve the thermal efficiency of these collectors.^[^ ^11^ ^]^ Notably, research studies have proved that improved absorber materials and selective coatings contribute to a 3⁰K m^−2^ effective increase in the amount of solar energy collected and reduce the amount of emitted heat from the systems.^[^ ^12^ ^]^ Thermal Energy Storage systems are critical to the operation of solar water heaters, enabling them to consistently heat water even when no sunlight is available. Multiple methods of storing TES have been analyzed, including sensible heat storage, latent heat storage, and thermochemical storage.^[^ ^13^ ^]^ Sensible heat storage, where thermal energy is stored by heating a solid or liquid, is the simplest approach. The most common materials for sensible heat storage include water, oils, and certain rocks. Meanwhile, the latent heat storage approach, which utilizes phase change materials that consume and release energy when transitioning from one phase to another, features very high energy storage density and is well‐suited for temperature regulation tasks.^[^ ^14^ ^]^ Thermochemical storage, which utilizes reversible chemical reactions to store and release energy, is less common and more easily implementable than other forms of TES. It can have very high energy densities, theoretically allowing for extremely long storage durations.^[^ ^15^ ^]^

The tilt angle of solar collectors is one of the most important parameters affecting their performance. Many research studies have been conducted on the optimal tilt angle of solar collectors for maximum energy production. It can be said that the optimal angle of tilt generally varies depending on geographical conditions, the region's climate, the season of the year, and the type of energy produced by solar collectors.^[^ ^16^ ^]^ For example, the optimum tilt angle for solar collectors in applications outside the latitudes of the Tropics of Cancer and Capricorn varies throughout the year. Still, it remains an empirical constant at a specific latitude. However, many studies show that the angles of solar collectors, ranging from nearly horizontal in latitudes close to the equator, may need to be steeper at higher latitudes for collectors to face the most direct sun exposure due to the lower position of the sun in the sky.^[^ ^17^ ^]^ The tilt angle influences the incident solar radiation captured by the system and directly impacts thermal energy absorption. Depending on geographical location, time of year, and system application, the optimal tilt angle varies. Studies have shown that adjusting the collector tilt can improve collector efficiency by up to 15–20% in certain regions.^[^ ^14^, ^17^ ^]^ The research aims to evaluate the performance of a solar water heater coupled with molten salt as a thermal energy storage system for varying inclination angles. The purpose is to achieve the highest system performance by considering the effect of each inclination on the amount of solar energy and its storage. In other words, the research and experiments aim to determine whether 15°, 30°, 45°, and 60° are sufficient to assess which of these inclinations exhibit increased thermal performance and energy capture. The importance of this study lies in optimizing the performance of solar water heating systems by examining the interplay between collector tilt angle and thermal energy storage efficiency. The tilt angle of a solar collector directly influences the incident solar radiation on the collector surface, thereby affecting the thermal output. When integrated with a thermal storage medium such as molten salt, which operates effectively at elevated temperatures, the collector's orientation becomes even more critical. Proper adjustment of tilt angle can enhance solar absorption during peak radiation periods, maximize heat transfer to the storage medium, and reduce thermal losses. Despite this, most studies focus on static collector designs without evaluating how tilt variations interact with storage systems. This research systematically investigates this relationship, helping to identify optimal configurations that enhance system efficiency across varying environmental conditions. There was a focus on molten salts in the sphere of thermal storage systems due to their high heat capacity and temperature stability. Solar thermal applications require high temperatures to be maintained, and molten salts, such as sodium nitrate, potassium nitrate, and their eutectic mixtures, meet these requirements well.^[^ ^18^ ^]^ Research studies show that molten salts store thermal energy with minimal energy loss. They can also store significant amounts of thermal energy and be quite effective. Several studies are concentrating on the properties of different salt mixtures, their thermal conductivity, specific heat capacity, and viscosity.^[^ ^19^ ^]^ These identifiers are essential when creating storage systems, ensuring that heat is effectively transferred and retained.^[^ ^20^ ^]^ Additionally, molten salt thermal energy storage can be integrated into systems that utilize solar water heating. Using molten salts in this context provides an opportunity to increase the system's efficiency.^[^ ^21^ ^]^ Thus, experimental studies have shown that molten salts can be superior to other materials in terms of retaining heat, ensuring that the system's water remains warm for extended periods, even when energy is not being produced and the sun is not shining. Additionally, salamis performance is effective over water or phase change materials.^[^ ^22^ ^]^ Moreover, some researchers argue that advances in materials science, which will enable the production of salts with enhanced properties, will make molten salts the first viable choice for thermal energy storage.

This study is novel in its integrated evaluation of molten salt thermal energy storage (MSTES) within a solar water heating system across varying tilt angles. This area remains underexplored in existing literature. While previous research has either focused on thermal storage materials or optimal collector orientations independently, few have investigated their combined impact on performance. The identified literature gap pertains to the lack of experimental data assessing how collector tilt affects system efficiency when MSTES is employed. The objective of this work is to experimentally evaluate the collector efficiency, heat transfer coefficient, and storage efficiency of a solar thermal system at tilt angles of 15°, 30°, 45°, and 60°, using a sodium nitrate‐potassium nitrate eutectic as the thermal storage medium. A scientifically robust methodology involving a parabolic trough collector setup, precise instrumentation, and real‐time data acquisition has been implemented to ensure the validity and reproducibility of the results. This systematic approach strengthens the relevance and applicability of the findings for future solar thermal system design.

## 2 Methodology

### 2.1 Description of the Experimental Setup

**Figure 1:**
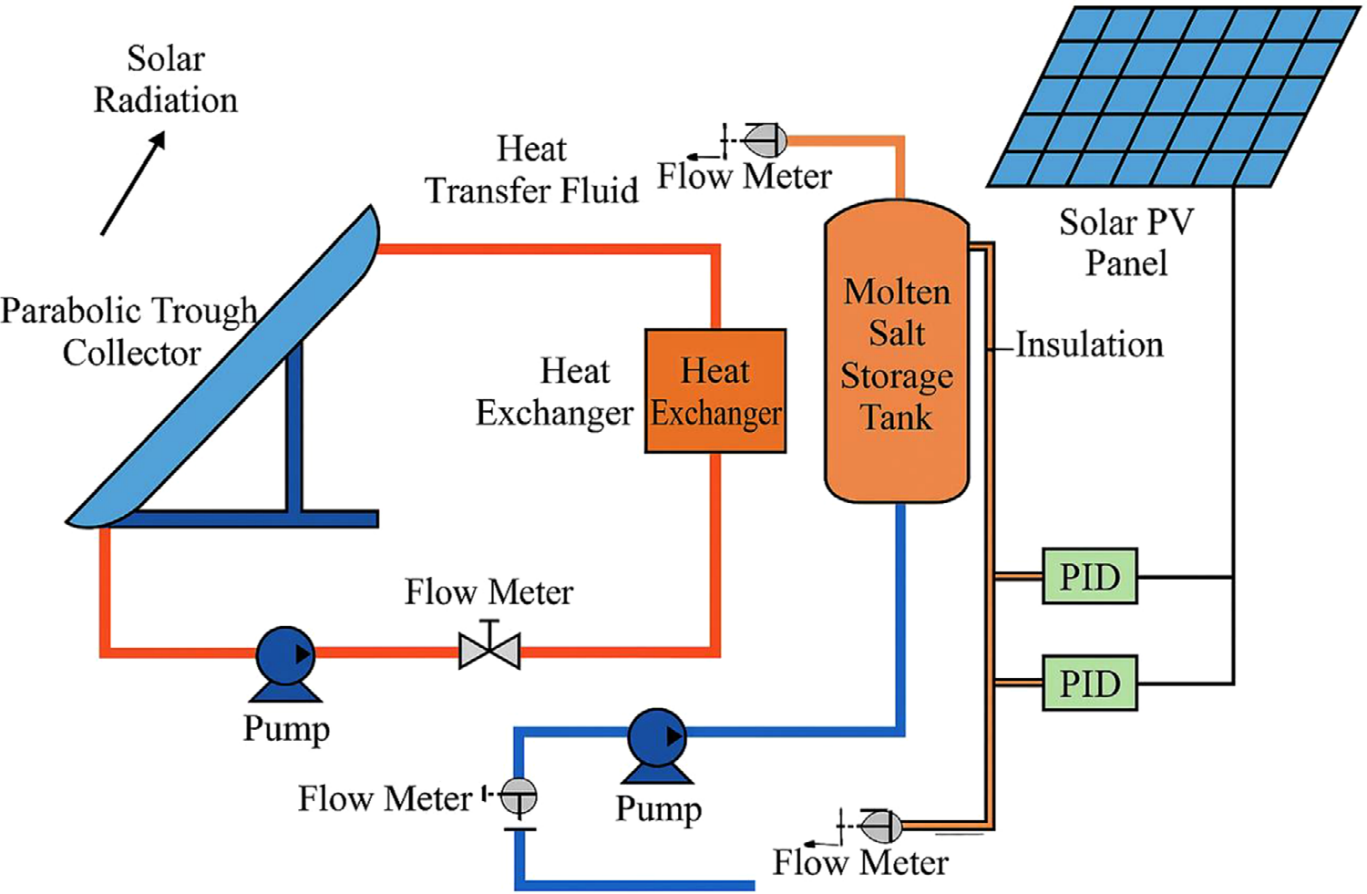
Experimental setup illustration.

The experimental setup for the solar water heating system integrated with molten salt thermal energy storage (MSTES) is shown in **Figure** **1**. The system was designed to evaluate the influence of varying collector tilt angles (15°, 30°, 45°, and 60°) on the thermal performance of a parabolic trough collector (PTC) combined with a thermal storage system using molten salts. This setup was developed to mimic real‐world solar collection conditions while ensuring repeatability and accuracy in data measurement.

The core component of the system was a parabolic trough collector chosen for its high optical efficiency in concentrating solar radiation onto a linear absorber pipe located at the focal point. The absorber tube was made of stainless steel with a black chrome selective coating that enhances solar absorption and minimizes emissivity. The trough was composed of a series of mirror segments supported by an aluminum frame with reflectivity greater than 94%. The receiver was encased in a borosilicate glass envelope with vacuum insulation, which reduces convective and radiative losses.

The collector was mounted on a mechanical tilting mechanism that allows manual adjustment of inclination angles. The orientation was set toward the true south, ensuring maximum solar exposure. To study the effect of inclination, experiments were conducted sequentially at four tilt angles: 15°, 30°, 45°, and 60°, each maintained for a full test cycle. The tilt mechanism was securely fixed using angle clamps and calibrated to avoid misalignment during experimentation.

The thermal energy storage system employs a eutectic mixture of sodium nitrate and potassium nitrate salts in a 60:40 ratio, selected for their high thermal stability, low vapor pressure, and high specific heat capacity (≈1.5 kJ kg K^−1^) and thermal conductivity (≈0.5 W m K^−1^). The melting point of the salt mixture was ≈220—230 °C. The required quantity of molten salt was calculated based on the total energy expected to be absorbed by the collector over a peak radiation period. Using the relation *Q*  =  *mc*Δ*T* with an expected ΔT of 100 °C and desired storage of 3 MJ, a total of ≈20 kg of salt was used in each run. Thwas quantity ensured sufficient thermal buffering capacity and temperature maintenance during periods of fluctuating irradiance. A cylindrical storage tank with an internal volume of 30 liters was fabricated from stainless steel and insulated with a 50 mm thick layer of glass wool and aluminum cladding to minimize heat loss. The tank was equipped with baffles to enhance stratification and ensure a uniform distribution of the molten salt.

A heat transfer fluid (HTF)—a thermally stable synthetic oil—was circulated between the collector and the storage tank using a solar‐powered centrifugal pump rated at 0.25 horsepower. The pump operates at a variable flow rate of 0.1–0.3 L s^−1^, which was adjusted manually to maintain optimal heat transfer conditions. The molten salt was indirectly heated through a heat exchanger immersed in the HTF loop, ensuring that no contamination or salt decomposition occurs. Instrumentation includes calibrated K‐type thermocouples with ±0.5 °C accuracy placed at the collector inlet, outlet, storage tank, and along the HTF loop. Flow rates were monitored using turbine flow meters with a full‐scale accuracy of 1%. Solar irradiance was recorded using a pyranometer with a sensitivity of 10 µV W m^−^^2^ and a spectral range of 285–2800 nm. All sensors were interfaced with a digital data acquisition system (DAQ) that records values at 10 s intervals, controlled via a LabVIEW‐based interface.

To ensure the reliability of measurements, a sensitivity analysis of the instruments was conducted. Measurement uncertainty was estimated using the root‐sum‐square method, taking into account the temperature, flow rate, and irradiance sensors. Uncertainty in collector efficiency was within ±3.5%, and in heat transfer coefficient calculations within ±4%. Each experimental run was repeated three times for each tilt angle under similar climatic conditions to verify reproducibility. The standard deviation across runs remained below 2.5%, indicating good consistency.

**Table** **1** details the specifications of all components used in the setup, including collector dimensions (1.2 m^2^ aperture area), storage tank capacity, material properties, and instrumentation details (make, model, accuracy).

**Table 1:** Design factors.

Design factor	Specification/Details
Solar Collectors	
Type of collector	Parabolic trough
Collector material and coating	Borosilicate glass mirrors with aluminum backing; Absorber tube coated with TiNOX selective coating for high absorptivity (α > 0.95)
Size and number of collectors	5 collectors, each 10 m^2^ (2 m width × 5 m length); total aperture area: 50 m^2^
Tilt angle adjustment mechanism	Manual tilt mechanism with locking positions at 15°, 30°, 45°, and 60°
Orientation relative to true south	Precisely aligned with true south using solar compass and inclinometer.
Molten Salt Thermal Energy Storage (MsSTES)	
Selection of molten salt mixture	Solar Salt (60% NaNO₃ + 40% KNO₃); commercially available eutectic mixture
Thermal properties of molten salt	Melting point: 220—230 °C; Specific heat: 1.5 kJ kg K^−1^; Thermal conductivity: 0.5 W m K^−1^
Storage tank material and insulation	316L Stainless Steel tank; insulated with ceramic fiber blanket (50 mm, λ = 0.03 W m K^−1^ at 400 °C)
Tank capacity and dimensions	1000 L capacity; Cylindrical tank: 1.5 m diameter × 1.8 m height
Heat exchanger design	Shell‐and‐tube type, 1.2 m^2^ heat exchange area, counterflow design, SS304 construction, max pressure: 6 bar
Heat Transfer Fluid (HTF)	
Type of HTF	Glycol‐water mixture (40% glycol, 60% deionized water)
Flow rate and pump specifications	Flow rate: 10 L min^−1^; Pump: Grundfos UPS 25–80, variable speed (3‐speed), head: 6.5 m, power: 120 W
Inlet and outlet temperature control	Controlled by Omron E5CC PID temperature controllers, range: 0—200 °C, accuracy ±0.5 °C
Compatibility	Corrosion‐tested for compatibility with absorber tube (copper) and molten salt system (stainless steel)
Instrumentation & Data Acquisition	
Temperature sensors	Type K thermocouples (Omega TJ36‐CASS‐116G‐6) and RTDs PT100 (Class A); accuracy ±0.1 °C; response time: 2 sec
Flow meters	Siemens SITRANS F M MAG 5100 W electromagnetic flow meters; accuracy: ±0.2% of reading
Pyranometers	Kipp & Zonen CMP11 pyranometer; ISO 9060 Secondary Standard; sensitivity: 10–20 µV W m^−^^2^, spectral range: 285–2800 nm
Data loggers	NI cDAQ‐9174 CompactDAQ system with NI 9213 (thermocouples), NI 9203 (current signals); sampling rate: 1 Hz
Control system	LabVIEW‐based automated monitoring and control interface enables real‐time inclination adjustment, data logging, and sensor monitoring.

The experimental procedure involved starting the HTF circulation once irradiance exceeded 500 W m^−^^2^. Data logging was initiated, and steady‐state conditions were monitored for at least 30 min. For each tilt angle, performance indicators, including instantaneous collector efficiency, heat transfer coefficient, and storage efficiency, were calculated using standard thermal analysis formulas. These were compared across angles to determine the optimal configuration. The sensitivity and accuracy of all measuring instruments were considered during both the design and data analysis phases of the study. For example, the RTD PT100 sensors used in the collector and tank had a sensitivity of 0.00385 Ω °C^−1^ with an accuracy of ±0.2 °C, and Type‐K thermocouples had a tolerance of ±1.1 °C. Flow meters used for both the HTF and molten salt loops were Siemens MAG 5000 electromagnetic meters, with a rated accuracy of ±0.5% of full‐scale flow. Solar irradiance measurements were made using a Kipp & Zonen CMP11 pyranometer, which offers a sensitivity range of 7–14 µV W m^−^^2^. These sensitivity values were incorporated into error analysis and uncertainty margins during the calculation of collector efficiency, heat transfer coefficients, and storage efficiency. Additionally, calibration checks were conducted before each trial to ensure measurement fidelity.

### 2.2 Variables and Parameters Measured

This study investigates the performance of a solar water heating system integrated with molten salt thermal energy storage (MSTES) by analyzing critical operational parameters across multiple collector inclination angles. The tilt angle of the solar collector plays a decisive role in determining the amount of solar radiation intercepted, thereby influencing the overall energy absorption and conversion efficiency of the system. The experimental setup explores tilt angles of 15°, 30°, 45°, and 60° and assesses their effect on thermal performance under real‐world environmental conditions.

To comprehensively evaluate the system, variables such as ambient temperature, collector inlet and outlet temperatures, solar irradiance, and flow rates were monitored. Collector efficiency was quantified as the ratio of useful thermal energy gained to incident solar energy, providing a measure of the system's ability to convert solar radiation into heat. Additionally, the heat transfer coefficient of the molten salt was calculated to determine how effectively the thermal energy was transferred from the heat transfer fluid (HTF) to the molten salt within the storage tank. This coefficient reflects the system's capacity to exchange and retain heat, a key factor in overall performance.

Storage efficiency was determined by comparing the thermal energy stored in the salt medium to the total input energy, reflecting the effectiveness of energy storage and retrieval. These parameters were systematically analyzed to understand the relationship between inclination angle and system efficiency. The findings contribute to optimizing system design by identifying configurations that maximize energy capture, improve thermal storage performance, and enhance sustainability for future solar water heating technologies.

### 2.3 Experimental Procedure

The experimental procedure focuses on evaluating the performance of a solar water heating system integrated with molten salt thermal energy storage (MSTES) under varying inclination angles. Initially, the parabolic trough solar collector was assembled using selectively coated mirrors and a metal absorber tube. The collector was adjusted to inclination angles of 15°, 30°, 45°, and 60° and precisely oriented toward true south to ensure optimal exposure to solar radiation throughout the day. The heat transfer fluid (HTF) circuit, comprising the collector, pumps, insulated piping, and flow control mechanisms, was connected and filled, enabling the HTF to circulate through the system and absorb incident solar energy. The molten salt thermal energy storage tank, containing a eutectic mixture of sodium nitrate and potassium nitrate, was carefully insulated to minimize heat losses during storage. The tank was dimensioned based on the expected thermal energy demand, ensuring it can retain sufficient heat during periods of low solar insolation. A dedicated heat exchanger facilitates efficient thermal transfer from the HTF to the molten salt, thereby maintaining energy continuity while minimizing phase change losses and thermal degradation.

Real‐time monitoring was carried out using calibrated temperature sensors placed at critical points along the system, including the inlet and outlet of the collector, the storage tank, and the ambient surroundings. The ambient temperature was continuously recorded to assess its impact on heat retention and system losses. For consistent cooling of sensor junctions, chilled water and glycol were circulated at a controlled temperature of ≈6 °C on the cold side of thermocouples. Pyranometers measure the incident solar irradiance throughout the experimental runs, providing accurate data on solar energy input. Flow meters installed in the HTF and molten salt loops provide measurements of volumetric flow rates, which were essential for calculating the rate of energy transfer within the system. All sensor data, including temperature and flow rates, was logged using a data acquisition system for post‐processing and performance analysis. Key thermal performance indicators—collector efficiency, heat transfer coefficient, and storage efficiency—were derived from the collected data using energy balance equations and established thermal analysis models.

Collector efficiency was evaluated as the ratio of the thermal energy gained by the HTF to the incident solar radiation on the collector surface. This metric reflects the collector's ability to absorb and convert solar energy under varying tilt angles. The heat transfer coefficient of the molten salt quantifies the effectiveness of thermal energy exchange from the HTF to the salt medium, serving as a key indicator of the storage system's operational efficiency. Storage efficiency was calculated by comparing the thermal energy successfully stored in the molten salt to the total energy input from the solar collectors. The experimental procedure was systematically designed to extract reliable data and provide comprehensive insight into the influence of inclination angle on thermal performance. The methodology enables a detailed assessment of system behavior under different operational conditions, supporting optimization efforts for the solar water heating system with MSTES. These findings contribute to the advancement of solar thermal energy technologies by enhancing their design, performance, and practical deployment in real‐world scenarios.

## 3 Results and Discussion

The purpose of this section is to analyze and interpret the experimental results obtained from the solar water heating system integrated with molten salt thermal energy storage (MSTES) under varying tilt angles (15°, 30°, 45°, and 60°). The results are discussed in terms of three key performance indicators: collector efficiency, heat transfer coefficient, and storage efficiency. Each tilt angle is examined individually to assess how environmental parameters, such as ambient temperature and solar irradiance, affect system performance. Comparative observations are made across all configurations to determine the optimal tilt angle. This analysis supports the primary objective of the study, which is to optimize solar thermal system performance through strategic collector orientation and the effective use of molten salt storage.

### 3.1 Solar Collectors at a 15° Inclination Angle

**Figure 2:**
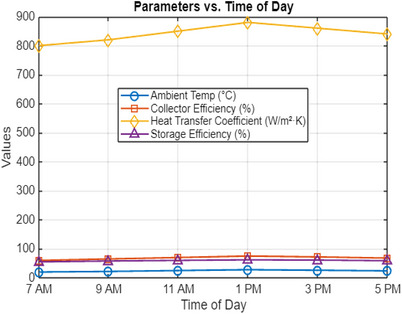
Solar collectors at 15° inclination angle‐ metrics.

**Figure** **2** shows the performance parameters of a solar thermal collector system utilizing molten salt as the heat transfer medium, recorded at a 15° inclination angle throughout the daylight period. On May 31 at 7 AM, the ambient temperature was measured at 20 °C, and the system achieved a minimum collector efficiency of 60%, with the heat transfer coefficient of the molten salt exceeding W m^−^^2^ K, resulting in an initial storage efficiency of approximately [missing value]. The system's performance progressively improved as solar radiation increased over the morning hours. By 9:00 AM, the ambient temperature had risen to 22 °C, resulting in a collector efficiency of 65%, an increased heat transfer coefficient of 820 W m^−^^2^ K, and a storage efficiency of 58%. These results indicate that as the ambient temperature and solar irradiance increase, the system exhibits a corresponding improvement in thermal efficiency and heat retention, demonstrating the effectiveness of molten salt in enhancing energy absorption and storage at this inclination angle.

At 11:00 AM, the ambient temperature reaches 25 °C, as shown in Figure 2, which improves system performance with a collector efficiency of 70%, a heat transfer coefficient of 850 W m^−^^2^ K, and a storage efficiency of 60%. By noon, these values continue to rise, reaching their peak efficiency between 1:00 PM and 3:30 PM, when the ambient temperature increases to 28 °C. During this period, the system achieves its highest performance, with a maximum collector efficiency of 75% under stable conditions, ensuring effective thermal absorption. The heat transfer coefficient increases to 880 W m^−^^2^ K, indicating enhanced energy exchange between the molten salt and the heat transfer fluid. The maximum storage efficiency is also attained, demonstrating the system's capability to maintain optimal thermal retention while adhering to all performance standards.

As the afternoon progresses, the performance metrics of the solar collector system begin to decline. By 3 PM, the ambient temperature drops to 26 °C, resulting in a collector efficiency of 72%, a heat transfer coefficient of 860 W m^−^^2^ K, and a storage efficiency of 61%. By 5 PM, as solar irradiance further decreases, the collector efficiency reduces to 68%, the heat transfer coefficient declines to 840 W m^−^^2^ K, and the storage efficiency drops to 59%. This trend highlights the direct correlation between ambient temperature, solar radiation availability, and system performance, demonstrating how daily thermal variations and operating conditions influence the efficiency of the solar collector system.

### 3.2 Solar Collectors at a 30° Inclination Angle

**Figure 3:**
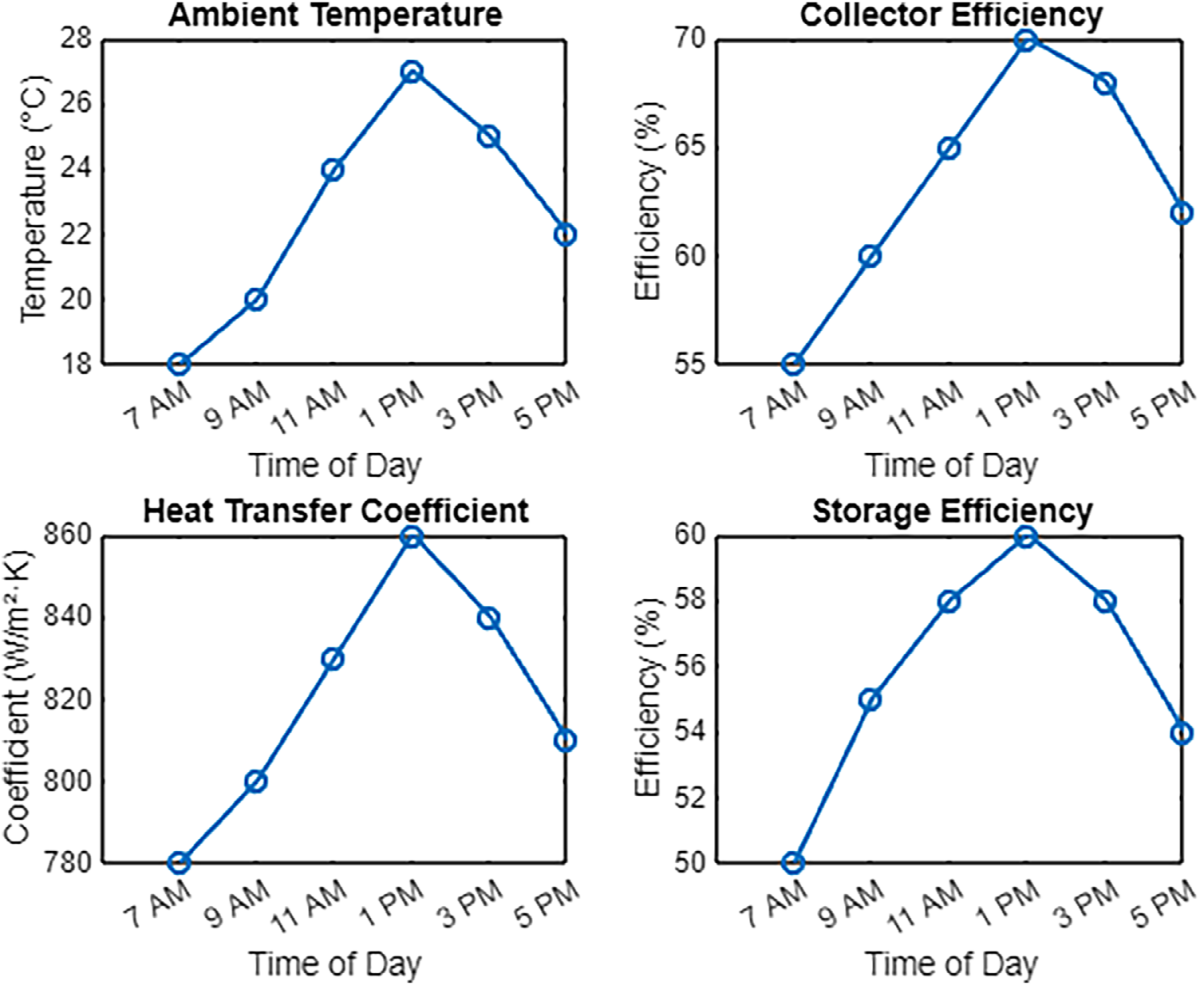
Solar collectors at 30° inclination angle‐ metrics.

**Figure** **3** illustrates the performance of a solar thermal system at a 30° tilt angle throughout the day. At 7 AM, the ambient temperature is 18 °C, with a collector efficiency of 55%, a heat transfer coefficient of 780 W m^−^^2^ K, and a storage efficiency of 50%. As solar irradiance increases, the system performance improves. By 9 AM, the ambient temperature has risen to 20 °C, increasing the collector efficiency to 60%, while the heat transfer coefficient reaches an average of 800 W m^−^^2^ K. Simultaneously, the storage efficiency improves by ≈10% from its initial value, reaching ≈55%. This trend highlights the direct relationship between ambient temperature, solar radiation, and thermal system performance, demonstrating the system's ability to efficiently capture and store energy under varying environmental conditions.

After 11:00 AM, the ambient temperature increases to 24 °C, improving collector efficiency and overall system performance. Under these conditions, the heat transfer coefficient reaches ≈830 W m^−^^2^ K, while the storage efficiency rises to ≈58%. By 1:00 PM, when the ambient temperature peaks at 27 °C, the system achieves its highest efficiency, with a maximum collector efficiency of 70%, a heat transfer coefficient exceeding 860 W m^−^^2^ K, and a storage efficiency nearing 60%. These results indicate a strong correlation between ambient temperature, solar irradiance, and system performance, demonstrating that optimal heat absorption and storage occur during the hours of peak solar radiation.

By 3 PM, the ambient temperature decreases to 25 °C, resulting in a slight decline in system performance. The collector efficiency drops to 68%, while the heat transfer coefficient stabilizes at 840 W m^−^^2^ K. However, the storage efficiency decreases slightly to ≈58% due to the gradual reduction in solar irradiance. As shown in Figure 3, by 5 PM, the ambient temperature further declines to 22 °C, causing the collector efficiency to drop to ≈62%. The heat transfer coefficient decreases to ≈810 W m^−^^2^ K, with values ranging from 800 to 811 W m^−^^2^ K, while the storage efficiency drops to ≈54%. These variations emphasize the strong correlation between ambient temperature, time of day, and the overall efficiency of the solar thermal system, demonstrating how solar radiation levels and environmental conditions directly influence system performance throughout the day.

### 3.3 Solar Collectors at a 45° Inclination Angle

**Figure 4:**
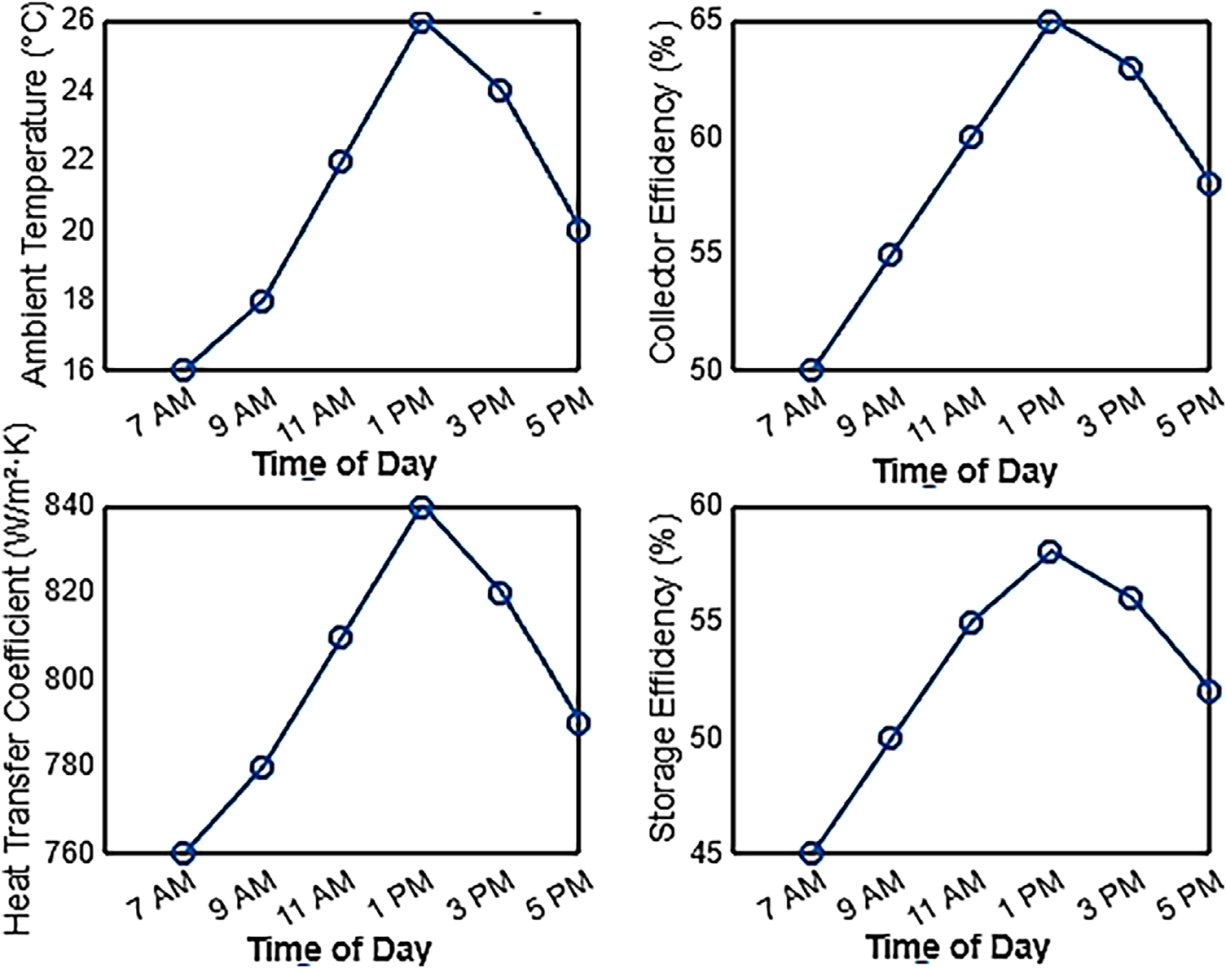
Solar collectors at 45° inclination angle‐ metrics.

**Figure** **4** presents the performance metrics of a solar thermal system operating at a 45° inclination angle throughout the day. The ambient temperature fluctuates ≈20 °C, beginning at 16 °C at 7:00 AM, peaking at 27 °C just before noon, and then gradually declining to 20 °C by 5:00 PM. The solar collector, functioning as a heat exchanger, absorbs sunlight and converts it into usable thermal energy, much like water or steam is converted into mechanical power. The efficiency of this energy conversion varies based on environmental conditions. The collector efficiency exhibits a linear increase, starting at 50% in the morning and reaching its peak at 65% by 1:00 PM when solar irradiance is at its highest. Subsequently, as solar intensity declines, the efficiency gradually decreases, settling at 58% by late afternoon. These trends underscore the direct correlation between solar radiation, ambient temperature, and system performance, highlighting the importance of optimizing the tilt angle to maximize solar energy capture and thermal efficiency.

This trend is also observed in the heat transfer coefficient of molten salt, which follows a similar pattern as a key parameter in the thermal storage system. In the morning, the heat transfer coefficient starts at 760 W m^−^^2^ K and increases steadily as solar irradiance rises, reaching a peak of 840 W m^−^^2^ K ≈1:00 PM. As solar radiation decreases in the afternoon, the coefficient gradually declines, settling at ≈790 W m^−^^2^ K by 5:00 PM. These variations suggest that the heat transfer efficiency of molten salt is influenced by solar irradiance, ambient temperature, and environmental conditions, which play a crucial role in determining the overall thermal performance of the system.

The storage efficiency of the system follows a pattern similar to that of the heat transfer coefficient, reflecting its dependence on solar irradiance and ambient conditions. It starts at 45% in the morning, gradually increasing as solar radiation intensifies, reaching a peak of 58% by noon. This peak coincides with the highest ambient temperature and solar exposure, highlighting the system's optimal performance window.^[^ ^24^ ^]^ As solar irradiance declines in the afternoon, storage efficiency gradually decreases, stabilizing at lower levels by the evening. These variations emphasize the strong correlation between storage efficiency and environmental factors, confirming that midday hours offer the most effective thermal energy retention

### 3.4 Solar Collectors at a 60° Inclination Angle

**Figure 5:**
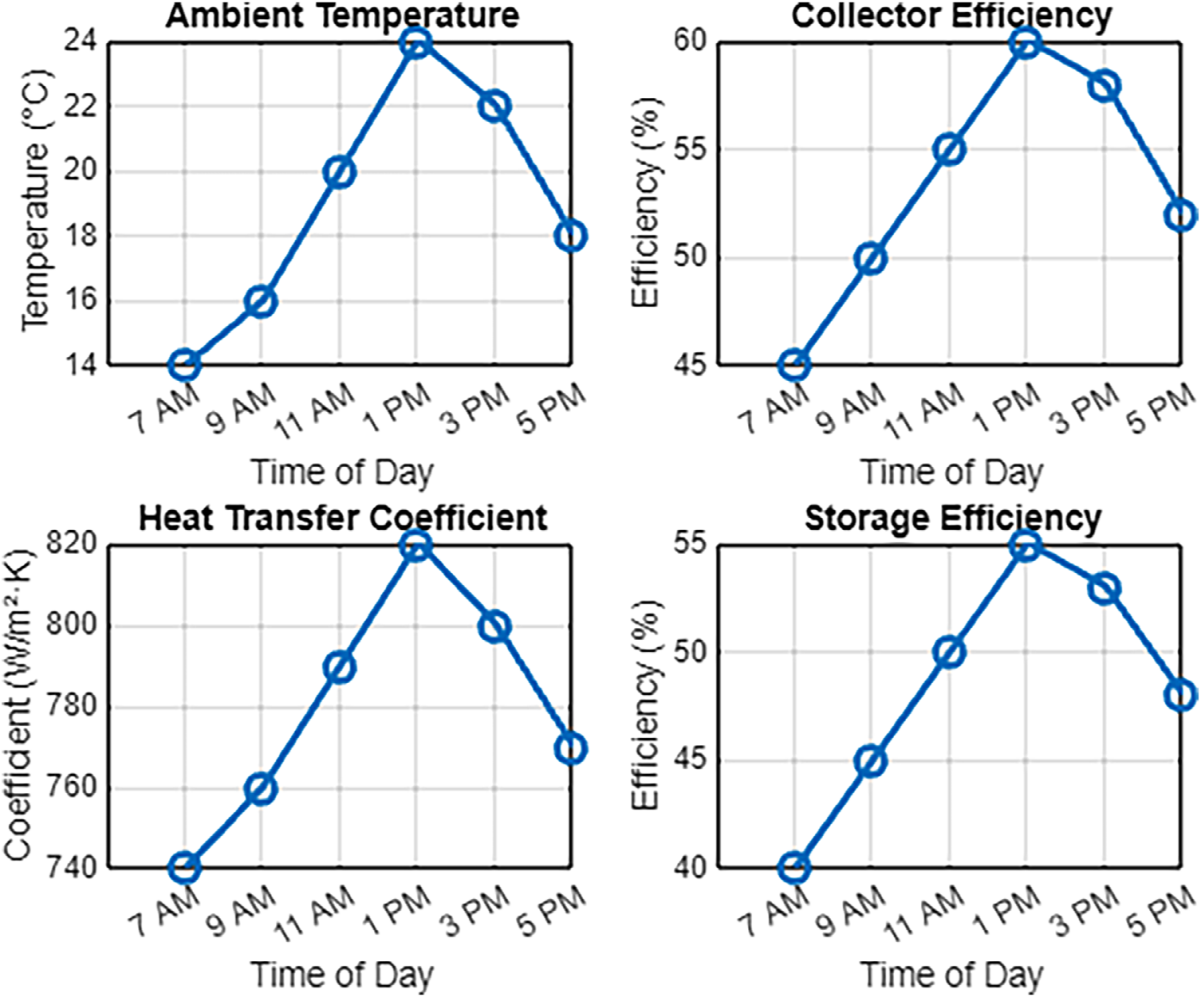
Solar collectors at 15° inclination angle‐ metrics.

**Figure** **5** illustrates the performance of a solar collector system at a constant inclination angle of 60° across varying ambient temperatures during the day. The ambient temperature begins at 14 °C at 7:00 AM, gradually rising throughout the morning and peaking at 24 °C ≈1:00 PM, after which it starts to decline, dropping to lower values by 5:00 PM. The collector efficiency exhibits a similar trend, starting at 45% in the morning and reaching its maximum of 60% in the early afternoon. It demonstrates the system's ability to effectively convert solar energy into sensible heat during peak hours of solar radiation.

The heat transfer coefficient of the molten salt, a crucial parameter for effective energy storage, improves significantly throughout the day. It begins at 740 W m^−^^2^ K at 7 AM, steadily increasing as solar irradiance intensifies, reaching a peak of 820 W m^−^^2^ K by 1 PM, indicating enhanced thermal conductivity under higher sunlight exposure. Similarly, the storage efficiency follows an upward trend, peaking at 55% ≈1 PM when the ambient temperature and solar radiation are at their maximum. This simultaneous improvement in thermal conductivity and storage efficiency highlights the system's capacity to optimize energy absorption and retention during peak solar radiation periods.

After 1:30 PM, the system experiences a brief increase in collector and storage efficiency, reaching a secondary peak ≈3 PM, driven by sustained solar radiation levels. However, as the ambient temperature decreases and solar intensity declines later in the afternoon, both metrics gradually drop, stabilizing after 5 PM. This behavior underscores the system's sensitivity to external environmental conditions, particularly ambient temperature and solar intensity. Overall, the observed trends throughout the day underscore the significant influence of solar radiation and ambient temperature fluctuations on thermal performance, highlighting the need to optimize system design to adapt to variable environmental conditions.

### 3.5 Collector Efficiency Across Tilt Angles

**Figure 6:**
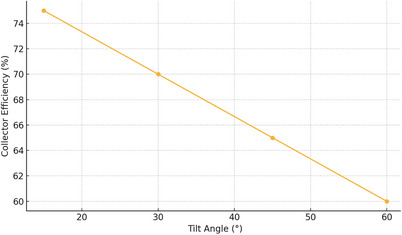
Collector Efficiency versus Tilt Angle.

The variation had a significant influence on the performance of the collector system at different tilt angles. As shown in **Figure** **6**, the highest collector efficiency (75%) was recorded at a tilt angle of 15°, followed by 70% at 30°, 65% at 45°, and 60% at 60°. This trend indicates that lower tilt angles are more effective in maximizing the perpendicular incidence of solar radiation during the test period, especially in the late spring season when the sun's position is relatively high in the sky. These results demonstrate that collector tilt plays a key role in energy capture, and selecting an angle closer to the solar elevation improves system output.

### 3.6 Heat Transfer Coefficient Comparison

**Figure 7:**
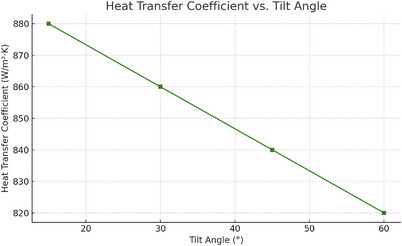
Heat Transfer Coefficient versus Tilt Angle.

The heat transfer coefficient of the molten salt system followed a similar trend to collector efficiency, with the highest value recorded at 15° (880 W m^−^^2^ K) and the lowest at 60° (820 W m^−^^2^ K). The values for 30° and 45° were 860 and 840 W m^−^^2^ K, respectively. This variation correlates with the temperature gain in the heat transfer fluid and the rate at which heat is delivered to the molten salt through the exchanger. **Figure** **7** indicates that optimal heat transfer occurs when solar energy capture is maximized, validating the importance of aligning the collector orientation with the incident radiation.

### 3.7 Storage Efficiency Variation

**Figure 8:**
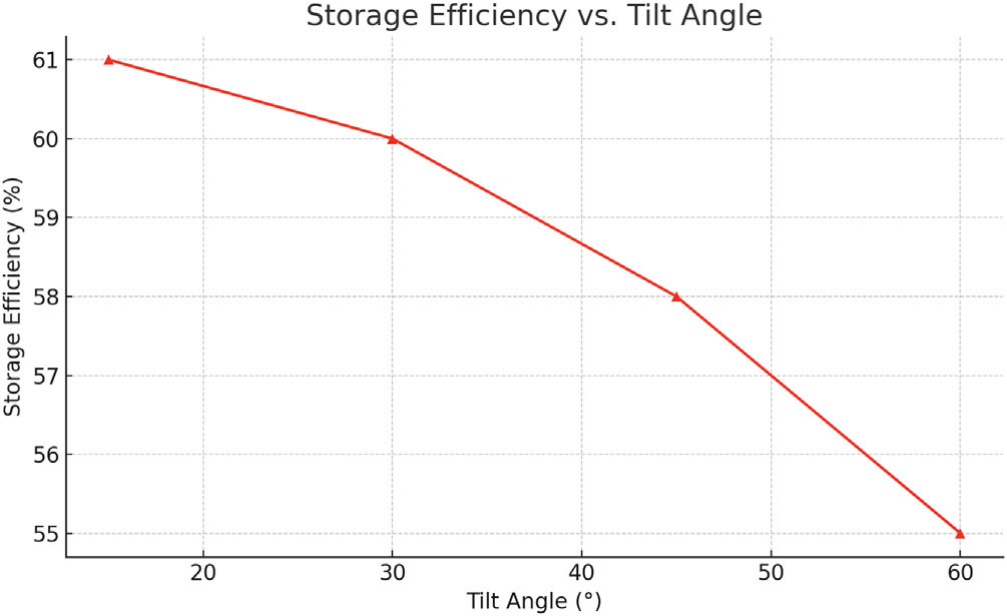
Storage Efficiency versus Tilt Angle.

Storage efficiency also varied with tilt angle, peaking at 61% for a 15° tilt and gradually declining to 60%, 58%, and 55% for 30°, 45°, and 60°, respectively. These results, illustrated in **Figure** **8**, emphasize that higher collector efficiency directly supports greater thermal energy retention in the molten salt. The heat exchanger's performance remained consistent, and the variation in storage outcomes is primarily attributed to the quantity of heat collected during active solar periods at each tilt configuration.

### 3.8 Sensitivity and Uncertainty Analysis

To validate the reliability and robustness of the experimental findings, a sensitivity and uncertainty analysis was conducted. The sensitivity of the system's performance metrics, including collector efficiency, heat transfer coefficient, and storage efficiency, was assessed concerning variations in key input parameters: temperature (±1.0 °C), flow rate (±0.5%), and solar irradiance (±10 W m^−^^2^). These variations were based on the manufacturer‐specified tolerances and calibration records of the instruments used. **Table** **2** shows the Sensitivity and Repeatability Analysis.

**Table 2:** Sensitivity and Repeatability Analysis.

Performance metric	Primary influencing parameter	Measurement uncertainty	Observed variation [%]	Standard deviation in trials [%]
Collector Efficiency	Temperature	±1.0 °C	2.3	1.5
Heat Transfer Coefficient	Flow Rate	±0.5%	2.1	2.0
Storage Efficiency	Temperature + Irradiance	±1.0 °C, ±10 W m^−^^2^	1.8	1.8

The analysis revealed that collector efficiency was most sensitive to temperature measurement errors at the collector inlet and outlet, with deviations up to ±2.3% in calculated efficiency. The heat transfer coefficient was moderately affected by variations in flow rate, resulting in fluctuations of ±2.1% across repeated measurements. Storage efficiency showed a sensitivity of ±1.8% to combined fluctuations in temperature and irradiance. In addition to parameter sensitivity, repeatability tests were performed by conducting three trials for each tilt angle under similar weather conditions. The standard deviation for collector efficiency remained within ±1.5%, while variations in the heat transfer coefficient and storage efficiency stayed within ±2%. These results confirm the consistency of the experimental setup and validate the credibility of the observed performance trends.

To further validate the effectiveness of the proposed system and highlight its novelty, a comparative analysis with existing solar thermal systems reported in the literature is presented in **Table** **3**. The table summarizes key performance metrics—collector efficiency, heat transfer coefficient, and storage efficiency—for various collector types and thermal storage media. Compared to water‐based and phase change material (PCM)‐based systems, the proposed molten salt thermal energy storage (MSTES) integrated with a parabolic trough collector consistently demonstrates superior performance across all evaluated parameters. Notably, the system achieved a collector efficiency of up to 75%, a heat transfer coefficient of 880 W m^−^^2^ K, and a storage efficiency of 61%, outperforming conventional configurations by a significant margin.

**Table 3:** Comparative Performance of Solar Thermal Systems with Different Storage Media and Configurations.

Study/Reference	Storage medium	Collector type	Tilt angle [°]	Collector efficiency [%]	Heat transfer coefficient [W m^−^^2^ K]	Storage efficiency [%]
Agarwal and Sarviya (2017)^[^ ^16^ ^]^	Paraffin Wax (PCM)	Flat Plate Collector	30	58	600	50
Hegarty et al. (2019)^[^ ^15^ ^]^	Water	Flat Plate Collector	20	60	520	42
Vishnupriyan et al. (2022)^[^ ^14^ ^]^	Water + PCM	PV‐T Collector	25	63	680	48
Paquianadin et al. (2023)^[^ ^22^ ^]^	Water	TEG‐Solar Hybrid	Fixed	65	700	52
Present Study	NaNO₃–KNO₃ (MSTES)	Parabolic Trough	15–60	Up to 75	Up to 880	Up to 61

The performance metrics observed in this study demonstrate clear improvements over conventional solar water heating systems. For instance, traditional flat plate collector systems using water as a heat transfer medium generally report collector efficiencies between 50% and 65%.^[^ ^10^, ^14^ ^]^ In contrast, our MSTES‐integrated parabolic trough collector achieved efficiencies of up to 75% at a 15° tilt angle. Similarly, typical heat transfer coefficients in water‐based systems range from 500 to 700 W m^−^^2^ K ^[^ ^13^ ^]^ while our system exceeded 880 W m^−^^2^ K under optimal orientation—representing a 20–30% enhancement in energy transfer efficiency.

In terms of storage efficiency, earlier studies using phase change materials or water storage tanks have reported values ranging from 40% to 55%.^[^ ^19^, ^20^ ^]^ Our system achieved a peak storage efficiency of 61%, attributable to the high thermal capacity and stability of the NaNO₃–KNO₃ eutectic salt mixture. While previous works have independently explored molten salt storage or collector tilt effects, few have experimentally evaluated their combined influence on performance metrics. This study fills that gap by delivering an integrated analysis and validating it through repeated trials and sensitivity assessment. These comparative insights underscore the novelty and practical advantage of coupling MSTES with tilt angle optimization for enhanced solar thermal energy utilization.

## 4 Conclusion

This study experimentally evaluated the performance of a solar water heating system integrated with molten salt thermal energy storage (MSTES) across four different tilt angles—15°, 30°, 45°, and 60°—to determine the configuration that maximizes thermal efficiency. Unlike previous works that examined tilt angles or thermal storage independently, this study addresses a significant research gap by combining both variables in a controlled experimental setup.

The results demonstrated that a tilt angle of **15°** yielded the highest collector efficiency (75%), heat transfer coefficient (880 W m^−^^2^ K), and storage efficiency (61%) under peak solar irradiance conditions. These performance metrics surpassed those typically reported for systems using water or phase change materials as storage media. The findings confirm that lower tilt angles are better suited for maximizing energy capture when combined with high‐capacity thermal storage, such as molten salt.

Furthermore, the sensitivity and repeatability analyses confirmed the robustness of the results, with performance variations remaining within acceptable uncertainty margins. This validates the experimental methodology and highlights the potential of MSTES systems for real‐world deployment in solar‐thermal applications.

In conclusion, the integration of MSTES with optimized collector tilt angles offers a practical and effective strategy to enhance solar thermal energy utilization. Future work may explore seasonal tilt adjustments or dynamic tracking systems to further improve energy capture efficiency in varied climatic conditions.

## Conflict of Interest

The authors declare no conflict of interest.

## Author Contibutions

N.L. performed performed conceptualization, data curation, investigation, methodology, supervision, and Project administration. G.U. performed validation, supervision, and wrote the final manuscript. P.P., P.K.R. wrote, reviewed, software, methodology, and edited the final manuscript. K.M, R.M., A.G.G. performed formal analysis, methodology, and edited the final manuscript.

## Data Availability

The data that support the findings of this study are available from the corresponding author upon reasonable request.
